# The impact of helmets on motorcycle head trauma at a tertiary hospital in Jamaica

**DOI:** 10.1186/1756-0500-2-172

**Published:** 2009-08-29

**Authors:** Ivor W Crandon, Hyacinth E Harding, Shamir O Cawich, Morton AC Frankson, Georgiana Gordon-Strachan, Noel McLennon, Archibald H McDonald, Doreen Fearon-Boothe, Nicole Meeks-Aitken, Karen Watson-Jones, Kenneth C James

**Affiliations:** 1 The Department of Surgery, Radiology, Anaesthesia and Intensive Care, The University Hospital of the West Indies, Mona, Kingston 7, Jamaica, W.I; 2 Office of the Dean, Faculty of Medical Sciences, The University of the West Indies, Mona, Kingston 7, Jamaica, W.I; 3 Department of Community Health and Psychiatry, The University of the West Indies, Mona, Kingston 7, Jamaica, W.I

## Abstract

**Background:**

Although the Jamaica road traffic act mandates motorcycle riders to wear approved helmets, opponents suggest that the local road conditions obviate any benefits from helmet use that have been proven in Developed countries. They suggest that the narrow, winding, poorly surfaced, congested local highways do not allow motorcyclists to sustain high velocity travel. The accidents then tend to occur at lower speeds and are accompanied by less severe injuries. This study was carried out to determine the impact of helmet use on traumatic brain injuries from motorcycle collisions in patients admitted to a tertiary referral hospital in Jamaica.

**Methods:**

A prospectively collected trauma registry maintained by the Department of Surgery at the University Hospital of the West Indies in Jamaica was accessed to identify all motorcycle collision victims from January 2000 to January 2007. The therapeutic outcomes of traumatic brain injuries were compared between helmeted and un-helmeted riders. The data was analyzed using SPSS Version 12.

**Results:**

Of 293 motorcycle collision victims, 143 sustained brain injuries. There were 9 females (6.3%) with an average age of 23 +/- 7.3 years and 134 males (93.7%) at an average age of 33.4 +/- 11.2 years (mean +/- SD). Only 49 (34.3%) patients wore a helmet at the time of a collision. Helmet use at the time of a collision significantly reduced the severity of head injuries (28.6% vs 46.8%, P = 0.028) and the likelihood of sustaining intra-cranial lesions (26.5% vs 44.7%, P = 0.03) from head injuries.

**Conclusion:**

Wearing a helmet at the time of a motorcycle collision reduces the severity of head injuries. However, the prevalence of helmet use at the time of a collision is unacceptably low.

## Background

Motorcycles are small lightweight performance oriented vehicles that have become a popular means of transportation. Compared to traditional automobiles, motorcycles are easier to maneuver, consume less fuel and have shorter acceleration and transit times [1]. These advantages come at the expense of exposure to weather and reduced passenger/cargo capacity. Riders are also at an increased risk of collision because the small motorcycle size makes them less conspicuous to automobile drivers [1].

When collisions occur, motorcycle riders are seriously disadvantaged by the lack of available safety equipment such as seat belts and air bags. An approved safety helmet is the single most important safety measure to protect motorcycle riders involved in collisions [1-6]. Helmeted riders have been shown to have 70% reduction in injury severity [2] and 40% reduction in mortality compared to un-helmeted riders in collisions [2,3]. Apart from protecting riders, the associated reduction in health care costs surrounding these events are obvious societal benefits [4,5].

Motorcycle users account for 12.6% of all road traffic accident victims requiring admission to the University Hospital of the West Indies (UHWI) in Kingston, Jamaica [7]. Although the Jamaica road traffic act mandates motorcycle riders to wear approved helmets [8], adversaries argue that the benefits of helmet use proven in Developed nations are not applicable to Jamaica because of the differing road conditions. This study was carried out in order to determine the impact of helmet use on traumatic brain injuries (TBI) from motorcycle collisions in patients admitted to the UHWI.

## Methods

The UHWI is a 500-bed tertiary hospital in that serves an estimated population of 826,880 persons living in and around Kingston, the capital of Jamaica [9]. The UHWI is equipped with seven operating theatre suites and two eight-bed multidisciplinary Intensive Care Units (ICU). Patients involved in road traffic accidents usually present to the emergency room, where they are evaluated, resuscitated and treated appropriately for their injuries.

The Department of Surgery at the UHWI maintains a trauma registry database where demographic and clinical data are prospectively collected from all patients requiring admission for trauma. This database was accessed to identify all motorcycle road traffic accident victims who required admission and/or treatment for head injuries between January 1, 2000 and January 1, 2007. The determination of helmet use at the time of the collision was based on reports provided by police and/or paramedic personnel.

Pedestrians and automobile passengers who were involved in collisions with motorcycles were not included in this study. We also excluded patients in whom helmet use could not be ascertained and those who did not suffer a traumatic brain injury (TBI).

The standardized TBI definition proposed by the Head Injury Interdisciplinary Special Interest Group of the American Congress of Rehabilitation Medicine was employed in this study [10]. A TBI was considered present if there was physiological disruption of brain function as evidenced by one or more of the following features: loss of consciousness for any duration; amnesia for the event; altered mental state; permanent or transient neurological deficits. The TBI was considered mild if there was a Glasgow Coma Score (GCS) ≥ 13, moderate if there was a GCS between 9-12 and severe if there was a GCS ≤ 8. An intracranial lesion was considered present if there was clinical or CT evidence of compound skull fractures, intra-cranial bleeding, parenchymal contusions, lacerations and/or oedema.

The following data were extracted and entered in a Microsoft Excel^® ^worksheet: patient demographics, helmet use, injury details, GCS at admission, Injury Severity Score (ISS) at admission, duration of hospitalization, mortality and Glasgow Outcome Scale (GOS) scores upon discharge. The Statistical Package for Social Sciences (SPSS) version 12.0 was used to compare data between helmeted and un-helmeted riders, using Chi squared tests of homogeneity and Fisher's exact tests to determine significance. A P Value ≤ 0.05 was considered statistically significant.

## Results

During the study period, 293 patients required hospital admission for injuries sustained in motorcycle collisions. The patients were predominantly males, outnumbering females by a ratio of 25:1. The use of helmets could not be ascertained in 23 cases and a further 127 patients did not sustain a TBI. These patients were excluded from further analysis.

The remaining 143 (53%) patients were diagnosed with TBI. Most of the patients were in the third and fourth decades of life. There were only 49 (34.3%) patients wearing a helmet at the time of the collision.

There were 9 females (6.3%) with an average age of 23 +/- 7.3 years (Mean +/- SD; Range 14-37; Median 20; Mode 19). These women were all pillion passengers. The only female pillion rider wearing a helmet was a 14 year old child, while the remaining 8 (88.9%) women did not have helmets at the time of collision.

There were 134 males (93.7%) at an average age of 33.4 +/- 11.2 years (mean +/- SD; Range 7-71; Median 32; Mode 26). Of this, 86 (64.2%) were un-helmeted riders at a mean age of 31.4 +/- 10.1 years (range 7-55; median 30; mode 30). There were 48 (35.8%) helmeted riders at a mean age of 37.1 +/- 12.1 years (range 18-71; median 38; mode 38). Only 10 (7.5%) males were pillion riders and none of them wore helmets.

Table 1 compares the presenting features and outcomes of helmeted and un-helmeted motorcycle collision victims.

**Table 1:** A Comparison of Head injuries in Un-Helmeted and Helmeted Motorcycle Collision Victims

**Parameter**	**Road Traffic Accident Victims**			P Value*	**OR, CI**
			
	Total	Un-Helmeted	Helmeted		
			
Total number of head injured patients	143	94	49	-	-
Intracranial Lesions	55	42 (44.7%)	13 (26.5%)	0.03	2.24, 1.05 - 4.75⊕
					
Brain Injury Severity					
Severe TBI	58	44 (46.8%)	14 (28.6%)	0.028	2.31, 1.08 - 4.89⊕
Moderate TBI	7	5 (5.3%)	2 (4.1%)	0.695	1.83, 0.34 - 10.04
Mild TBI◆	78	45 (47.9%)	33 (67.3%)	1.000	1.0, -
					
ICU Admission	10	8 (8.5%)	2 (4.1%)	0.49	2.19, 0.45-10.72
					
ICU Duration of Stay					
Mean +/- SD		8.9 +/- 5.7	5.5 +/- 6.4		-
Range		4 - 21	1 - 10		
Median		7.5	5.5		
IQR (Q_1_, Q_3_)		4.25, 11.5	1, 10		
					
Injury Severity Score					
Mean +/- SD		15.5 +/- 15.0	13.3 +/- 22.1	0.143	-
Range		1 - 75	4 - 75		
Median		9	9		
Mode		9	4		
					
Hospitalization					-
Mean +/- SD		11.1 +/- 16.3	12.9 +/- 14.9	0.517	
Range		0 - 86	0 - 60		
Median		5	7		
IQR (Q_1_, Q_3_)		2, 11	2, 16.5		
					
Mortality	12 (8.4%)	10 (10.6%)	2 (4.1%)	0.22	2.80, 0.59 - 13.31
					
GOS Score Category					
Score ≤ 3	15 (10.5%)	13 (13.8%)	2 (4.1%)	0.071	3.77, 0.82-17.44
Score ≤ 2◆	128 (89.5%)	81 (86.2%)	47 (95.9%)	1.0	-

*Chi squared or fisher's exact test P values used as appropriate.

⊕ Statistically significant difference

◆ Reference category

The GOS was used as a predictive index of patient disability after discharge. The GOS scores were ≤ 2 in 128 (89.5%) patients, predicting functional independence in their daily living activities after discharge. There were 15 (10.5%) patients with GOS scores ≥ 3 that predicted serious disabilities. The prevalence of having a discharge GOS ≥ 3 became disproportionately greater when patients were un-helmeted (13.8%; 13/94) compared to those who wore helmets (4.1%; 2/49), although this did not achieve statistical significance.

Twelve (8.4%) patients died as a direct result of injuries sustained in the collision, and TBI was the cause of death in 9 (75%) of these cases. Eleven (91.7%) fatalities occurred in male motorcycle riders and 1 (8.3%) in a female pillion passenger. Ten of the twelve (83.3%) patients who died were not wearing helmets at the time of the collision.

## Discussion

The use of an approved safety helmet that meets US Federal Motor Vehicle Safety Standards (FMVSS) 218 is the single most important factor to significantly reduce the severity of head and neck injuries resulting from motorcycle collisions [1-6]. Helmeted riders involved in collisions have been shown to have approximately 70% reduction in injury severity [2] and 40% reduction in the risk of mortality [2,3]. The other proven benefits include a reduced likelihood of sustaining head injury, reduced requirement for and duration of ICU stay and significantly lowered health care costs associated with the treatment of these patients [4,5].

Despite fierce protest by anti-helmet lobbyists on the grounds of infringement of individual rights and freedoms [6,11], these data have been widely used to justify the introduction of laws mandating motorcycle riders to wear approved helmets [11,12]. In some instances the laws have been repealed, resulting in a notable increase in morbidity and mortality [6,12].

Adversaries of the helmet laws in Jamaica have suggested that the data showing reduced morbidity and mortality with the use of an approved helmet may not be applicable to local motorcycle riders. They cite road conditions that differ from developed countries where multiple lane, well maintained speedways allow sustained high velocity traffic. In contrast, the narrow, winding, poorly surfaced and congested local highways do not allow motorcyclists to sustain high velocity travel. While these conditions may not reduce the incidence of accidents, opponents claim that they tend to occur at lower speeds and are accompanied by less severe injuries. However, our data shows that helmet use still results in a significant reduction in the severity of TBI and intracranial lesions from head injuries.

Despite the existence of legislation to mandate motorcycle riders to use approved helmets [8], only a third of the patients sustaining TBI were helmeted at the time of a collision. While these figures are well below those reported from high income countries, they are comparable to reports from other developing nations [13-15]. This is a dire situation that demands urgent attention from law enforcement agencies.

The reasons for non-compliance were not specifically studied but may include the cost of the helmet, ignorance, a cultural disposition toward lawlessness, fatalism, insufficient educational campaigns, and/or recreational drug use, which has been associated with non-compliance [16]. A survey of un-helmeted riders revealed that 26% did not wear helmets because they were uncomfortable and inconvenient, and 53% simply had no expectation of accident involvement [1]. Other arguments that have been advanced in opposition to helmet use include impaired rider vision, attenuation of critical traffic sounds, rider fatigue and increased neck injuries in the event of a collision. However, none of these alleged disadvantages have been supported by evidence [1,3,14].

Educational campaigns to target these myths may be one way to increase helmet compliance. The World Health Organization recognized the importance of these statistics and has intensified efforts to support governments in low-income countries to increase helmet use [2,17]. The public educational campaigns may target young males by distributing this data via mass media.

As expected, helmet use did not have any significant effect on the duration of hospitalization because helmets offer no protection against trauma to the torso or extremities. Although the ISS has been shown to correlate linearly with mortality, morbidity and hospitalization [18,19], we did not expect a direct correlation because the ISS is an anatomical scoring system that evaluates only one injury from multiple body systems [18,19]. The relevance of the similarity in ISS is to show that helmeted and un-helmeted riders in this study were well matched as they were subjected to comparable levels of trauma [18,19].

The GOS is widely used as a reliable indicator of patient outcomes after TBI [20,21]. It is based upon the patient's ability to perform activities of daily living and the degree of assistance required [20]. Upon discharge from hospital, 90% of patients had GOS scores ≤ 2 and were able to function independently, resuming virtually all activities of daily living.

Only 15 patients had discharge GOS scores ≥ 3. At best, this predicts that the patients would no longer be capable of engaging in most personal, social or work activities due to limited communication skills, abnormal behavioral or unadjusted emotional responses [20,21]. Although there was a greater proportion of un-helmeted riders with GOS scores ≥ 3 (13.8% vs 4.1%, P = 0.071), this did not achieve statistical significance. However, this relationship holds clinical significance for these patients.

We cannot afford to waste resources, both health and human, which are consequent in treating unnecessary injuries and the resultant deaths, already of epidemic proportions in Jamaica [7,22]. Active (e.g. traffic regulations, education of riders) and passive measures (e.g. safety helmets) can be expected to reduce both the incidence and severity of head injuries among motorcyclists and need to be enforced.

## Conclusion

Wearing a helmet at the time of a motorcycle collision in this setting reduces severity of head injuries. However, the prevalence of helmet use at the time of a collision is unacceptably low. A campaign involving increased education and visible enforcement of the helmet law may be needed.

## Competing interests

The authors declare that they have no competing interests.

## Authors' contributions

IC conceived the study, participated in its design and drafted the manuscript. HH participated in the study design, statistical analysis and drafting of the manuscript. EW participated in data acquisition, and helped to draft the manuscript. SC participated in data acquisition, study design and drafting of the manuscript. JWJ participated in data acquisition and helped in drafting the manuscript. All authors have read and approved the final manuscript.
